# Psychological and Burnout Effects of the COVID‐19 Pandemic on Individuals

**DOI:** 10.1002/iid3.70181

**Published:** 2025-03-21

**Authors:** Bahar Ürün Ünal, Neslihan İyit, Yunus Akdoğan, Burcu Gök Erdoğan, Yüksel Duygu Altıparmak

**Affiliations:** ^1^ Department of Family Medicine Selcuk University Konya Turkey; ^2^ Department of Statistics Selçuk University Konya Turkey; ^3^ Department of Family Medicine Yeditepe University İstanbul Turkey

**Keywords:** burnout, confirmatory factor analysis, COVID‐19 pandemic, psychological distress, structural equation modelling

## Abstract

**Background:**

COVID‐19 infection has affected individuals mentally and socially in many areas. Restrictions, fear of infection, and anxiety about the future have created a great psychological burden on people. In this study, we aimed to investigate the significant negative effects of the COVID‐19 pandemic on individuals' mental health and psychological functioning.

**Methods:**

A 42‐question questionnaire was applied to a total of 557 participants, including 350 people over the age of 18 who applied to the COVID‐19 Vaccination Unit of Selçuk University Faculty of Medicine Hospital between December 2021 and May 2022 to be vaccinated and 207 people with a simultaneous online survey application. The psychological effects experienced by the participants due to the COVID‐19 epidemic were questioned with psychological distress and burnout scales. Differences in the demographic characteristics of the participants were investigated according to the fear and anxiety sub‐dimension.

**Results:**

According to the psychological distress scale, gender, COVID‐19 vaccination status, educational status and place of residence were found to be significant. According to the burnout scale, gender, marital status, presence of chronic disease, COVID‐19 posttest status, occupation and income status were found significant.

**Limitations:**

The anxiety, fear, and stress levels reported by participants may not be consistent with an objective assessment by mental health professionals. The majority of participants were public sector employees and students, so the results regarding job loss anxiety cannot be generalized. No information was collected on participants' past medical psychiatric disorders within the scope of the study.

**Conclusions:**

It is obvious that stress and psychiatric disorders are more common in individuals with high perception of infectiousness and lethality of the agent, especially during epidemic periods. We think that this study will be useful for planning interventions to alleviate mental health problems of individuals in future epidemics.

**Abbreviations**
BS: burnout scale
CFA: confirmatory factor analysis
D: doubt
FA: fear and anxiety
KMO: Kaiser Meyer Olkin
PDS: psychological distress scale
SEM: structural equation model
WHO: World Health Organization

## 1 Introduction

SARS‐CoV‐2 virus is an enveloped, single‐stranded, and positive‐sense RNA virus from the coronaviridae family. A pandemic was declared by the World Health Organization (WHO) on 11 March 2020 [1]. While some of the patients survived the disease with mild symptoms, some of the severe cases resulted in death. The COVID‐19 pandemic directly and indirectly affected the mental health of individuals and caused the emergence of various mental disorders in individuals [2].

The COVID‐19 pandemic, which has a high infection and mortality rate, has brought many important psychological problems, such as stress, anxiety, depression, fear, and burnout [1]. Stress factors such as restrictions, fear of infection, fear of infecting others, anxiety about the future, financial concerns, worry about loss of labour force and fear of death have seriously affected people psychologically. To prevent such psychological problems, understanding the factors associated with the psychological and burnout levels of individuals during the COVID‐19 pandemic is extremely important in terms of trying to determine the demographic characteristics of individuals and their strengths against the pandemic.

Recent studies on the COVID‐19 pandemic in the literature have shown that people experience many psychological problems such as stress, anxiety, worry, fear, frustration, and burnout during the COVID‐19 pandemic [2, 3, 4, 5, 6]. The severe experience of these psychological problems caused by the pandemic negatively affected people's social relations and led to major labour force losses [6, 7]. However, pandemics may cause exacerbation or persistence of psychological symptoms such as depression, anxiety and anxiety [2, 3, 4]. For this reason, the aim of this study was to investigate the significant negative effects of the COVID‐19 pandemic on individuals' mental health and psychological functioning by using the psychological distress scale and burnout scale.

This article was presented as an oral presentation at the 23. Eastern Mediterranean Family Medicine Congress held in Edirne on 9–12 May 2024.

## 2 Materials and Methods

### 2.1 Study Design

Between December 2021 and May 2022, a 20‐question questionnaire including sociodemographic data, COVID‐19 psychological distress scale consisting of 12 questions and COVID‐19 burnout scale form consisting of 10 questions were applied to 350 people who applied to the COVID‐19 Vaccination Unit of Selçuk University Faculty of Medicine between December 2021 and May 2022 and to 207 people simultaneously with an online survey.

The purposes and procedures of the study were appraised and approved by the ethics committee of our university before carrying out any relevant analyses (Decision no: 2021/512). All aims and means of the study were confirmed to be designed according to the ethical standards of the institutional research committee and the Helsinki Declaration and its later amendments. Approval was obtained from the Ethics Committee of Selçuk University Faculty of Medicine.

After the participants were informed about the study and their personal informed consent was obtained, the questionnaire form was applied. Illiterate, under 18 years of age and non‐volunteers were excluded from the study.

### 2.2 Sociodemographic Information Form

The form created by the researchers for sociodemographic data to be used in the study includes questions such as age, gender, education level, occupation, marital status, place of residence, household income, COVID‐19 history, smoking status, alcohol consumption, chronic diseases, and vaccination status of the participants.

### 2.3 Psychological Distress Scale

The psychological distress scale was developed by Feng et al. in 2020 [7]. Its Turkish validity and reliability was conducted by Ay et al. [8] The scale has a total of 12 items and has two sub‐dimensions. Items 1, 2, 3, 4, and 6 constitute the fear and anxiety dimension, while items 5, 7, 8, 9, 10, 11, and 12 constitute the doubt dimension. The five‐point Likert‐type scale is scored as “strongly agree (5)”, agree (4), “not sure (3)”, “disagree (2)”, “strongly disagree (1)”. There are no reverse items in the scale. The total score obtained from all items of the scale reflects the level of psychological distress experienced by the individual in relation to COVID‐19. The scores that can be obtained from the scale are between 12 and 60. The high score obtained from the scale means that there is a high level of psychological distress related to COVID‐19. Cronbach Alpha values of the sub‐dimensions of the scale were reported as 0.83 for the fear and anxiety dimension, 0.85 for the doubt dimension and 0.88 for the general psychological distress scale.

### 2.4 Burnout Scale

The burnout scale was developed by Malach‐Pines in 2005 [9]. Its Turkish validity and reliability was performed by Yıldırım and Solmaz in 2020 [10]. The scale has a total of 10 items and has a single dimension. The five‐point Likert‐type scale is scored as “always (5)”, “often (4)”, “undecided (3)”, “sometimes (2)”, “never (1)”. There are no reverse items in the scale. The total score obtained from all items of the scale reflects the level of burnout experienced by the individual in relation to COVID‐19. The scores that can be obtained from the scale are between 10 and 50. The high score obtained from the scale means that there is a high level of burnout related to COVID‐19. Cronbach Alpha values of the scale were reported as 0.92.

### 2.5 Statistical Analysis

Since the scales previously tested and used in the literature were utilized in the study, Confirmatory Factor Analysis was conducted to verify the scales and dimensions in question. Cronbach's Alpha coefficient was calculated for reliability analysis. Kolmogorov‐Smirnov test was used for the conformity of the data to normal distribution. Kruskal Wallis Test for more than two independent groups and Independent Two Group Mann‐Whitney U Test for two independent groups were used for data analysis. The interaction between variables was revealed by structural equation modelling. IBM SPSS 22 and IBM AMOS 22 package programmes were used in the analyses.

## 3 Results

A total of 557 participants, 387 women, and 170 men, were included in the study. The mean age of the participants was 47.17 ± 21.4 years (min‐max: 18–92). 61.2% of the participants were undergraduate graduates, 81.3% lived in the city centre, 52.2% were students, 39.7% had an income of 2000 TL or less, 81% were non‐smokers, 15.4% had chronic diseases, and 94.6% had at least one dose of vaccination (Table 1).

**Table 1:** Sociodemographic characteristics of the COVID‐19 pandemic participants in this study.

		Frequency	Percent (%)
**Gender**	Female	387	69.5%
	Male	170	30.5%
**Marital status**	Single	186	33.4%
	Married	371	66.6%
**Educational status**	Primary education	22	4.0%
	High school	46	8.3%
	Associate degree	23	4.1%
	Undergraduate	341	61.2%
	Graduate	125	22.4%
**Living place**	Province	453	81.3%
	District	84	15.1%
	Village	20	3.6%
**Profession**	Health employee	125	22.4%
	Small business	12	2.2%
	Student	291	52.2%
	Public sector	36	6.5%
	Private sector	41	7.4%
	Housewife	26	4.7%
	Not working	17	3.1%
	Worker	9	1.5%
**Income status**	Less than 2000 TL	223	40.0%
	2000–4000 TL	83	14.9%
	4000–6000 TL	68	12.2%
	6000–8000 TL	39	7.0%
	More than 8000 TL	144	25.9%
**Presence of chronic disease**	Yes	86	15.4%
	No	471	84.6%
**COVID‐19 vaccine status**	Positive	527	94.6%
	Negative	30	5.4%
**Sinovac dose (*n*** = **177)**	1	8	1.4%
	2	148	26.6%
	3	19	3.4%
	4	2	0.4%
**Biontech dose (*n*** = **456)**	1	49	8.8%
	2	334	60.0%
	3	71	12.7%
	4	2	0.4%
**Age**	28,42 ± 10,65 (min‐max: 16–69)		

Psychological distress scale was used as 12 items and two dimensions. The scale consists of two sub‐dimensions: fear, anxiety and doubt.

As the main findings of the confirmatory factor analysis (CFA) of the psychological distress scale used in the study, Kaiser‐Meyer‐Olkin (KMO) coefficient and Barlett's significance coefficient were determined as 0.865 and 0.000, respectively. According to these findings, it can be said that the sample size is sufficient for the study and the COVID‐19 pandemic data obtained through the questionnaire study are suitable for the application of the CFA method in this study. According to the goodness of fit values obtained as a result of CFA, the 5th and 7th questions of the “doubt” sub‐dimension were not confirmed and were removed from the study, and it is seen that the 2‐factor structure of the scale is good. The final scale consisting of 10 items met the validity criteria (*χ*²/Sd = 159,599/34 = 4,694; *p* < 0.00, CFI = 0.919; NFI = 0.900; RMSEA = 0.079). According to the CFA fit indices, it is seen that the fit indices are at acceptable and borderline acceptable values and the two‐dimensional structure of the psychological distress scale is confirmed.

The factor loadings of the sub‐dimensions of the psychological distress scale and the reliability results of each sub‐dimension of the scale are given in Table 2. As a result of the CFA analysis, the factor loads of the scale applied through the questionnaire were obtained between 0.545 and 0.784 for the fear and anxiety sub‐dimension of individuals with COVID‐19 vaccination at the workplace, and between 0.501 and 0.810 for the suspicion sub‐dimension. Therefore, it is seen that the factor loadings in this study were generally above 0.50. The Cronbach's alpha values of the psychological distress scale and total variance analysis (VA) 52,657 are given in Table 2 as 0.827 and 52.657, respectively. The reliability level of the psychological distress scale and its sub‐dimensions was high, Cronbach's alpha > 0.700.

**Table 2:** Reliability and factor loading results of psychological distress scale.

	Scale questions	Scale codes	Factor loadings	Cronbach's Alpha
**Fear and anxiety**	I worry when I see in the news that the number of COVID‐19 patients is increasing.	PDS3	0,784	0,758 VA:39,824
	I am afraid of travelling to a place with a high incidence of COVID‐19.	PDS2	0,700	
	I think that frequent visits to hospitals increase the risk of contracting COVID‐19.	PDS4	0,696	
	I think that frequent use of planes, trains, buses or other public transport increases the risk of contracting COVID‐19.	PDS6	0,664	
	When talking to someone I don't know, I suspect that they have COVID‐19.	PDS1	0,545	
**Doubt**	If I see someone without a mask, I suspect they have COVID‐19.	PDS11	0,810	0,757 VA:12,833
	If I see someone vomiting, I suspect they have COVID‐19.	PDS8	0,747	
	If I see someone coughing, I suspect they have COVID‐19.	PDS10	0,726	
	I suspect that new coronaviruses are in the air when someone is around.	PDS12	0,700	
	I am afraid of living near COVID‐19 isolation hospitals.	PDS9	0,501	
	VA = 52.657, Cronbach's Alpha = 0.827			

Abbreviation: VA, variance analysis.

Another scale used in the study is the burnout scale. The burnout scale developed by Malach‐Pines was used to determine the psychological distress levels of the participants. It consists of 10 items and one dimension. As a result of the CFA analysis of the burnout scale used for the study, the KMO coefficient and Barlett's significance coefficient were determined as 0.907 and 0.000, respectively. According to these findings, it can be said that the sample size is sufficient for the study and the COVID‐19 pandemic data obtained through the survey study is suitable for the application of the CFA method in this study.

According to the goodness of fit values of the CFA analysis applied to the COVID‐19 pandemic data, the 7th and 10th questions of the suspicion sub‐dimension were not confirmed and were removed from the study, and the 8‐item single‐factor structure of the scale meets the validity criteria (*χ*²/Sd = 98.718/20 = 4.936; *p* < 0.00, CFI = 0.966; NFI = 0.958; RMSEA = 0.080). It is seen that the fit indices related to the CFA analysis are at acceptable and borderline acceptable values and the burnout scale is confirmed.

The factor loadings and reliability results of the burnout scale are given in Table 3. As a result of the CFA analysis on individuals who received the COVID‐19 vaccine, the factor loadings of the scale applied through the questionnaire were obtained between 0.501 and 0.887. Therefore, it is seen that the factor loadings in the study were generally above 0.50. The reliability level of the burnout scale was determined to be high, Cronbach Alpha > 0.700.

**Table 3:** Reliability and factor loading results of the burnout scale.

	Scale questions	Scale codes	Factor loadings	Cronbach's Alpha
**Burnout scale**	How often do you feel trapped?	BS4	0,887	0,881
	How often do you feel helpless?	BS5	0,864	
	How often do you feel hopeless?	BS3	0,839	
	How often do you feel depressed?	BS6	0,813	
	How often do you feel worthless/unsuccessful?	BS8	0,716	
	How often do you feel tired?	BS1	0,679	
	How often do you feel disappointed in people?	BS2	0,653	
	How often do you have difficulty sleeping?	BS9	0,501	
	I suspect that new coronaviruses are in the air when there is someone around.	PDS12	0,700	
	I am afraid of living near COVID‐19 isolation hospitals.	PDS9	0,501	

**Figure 1:**
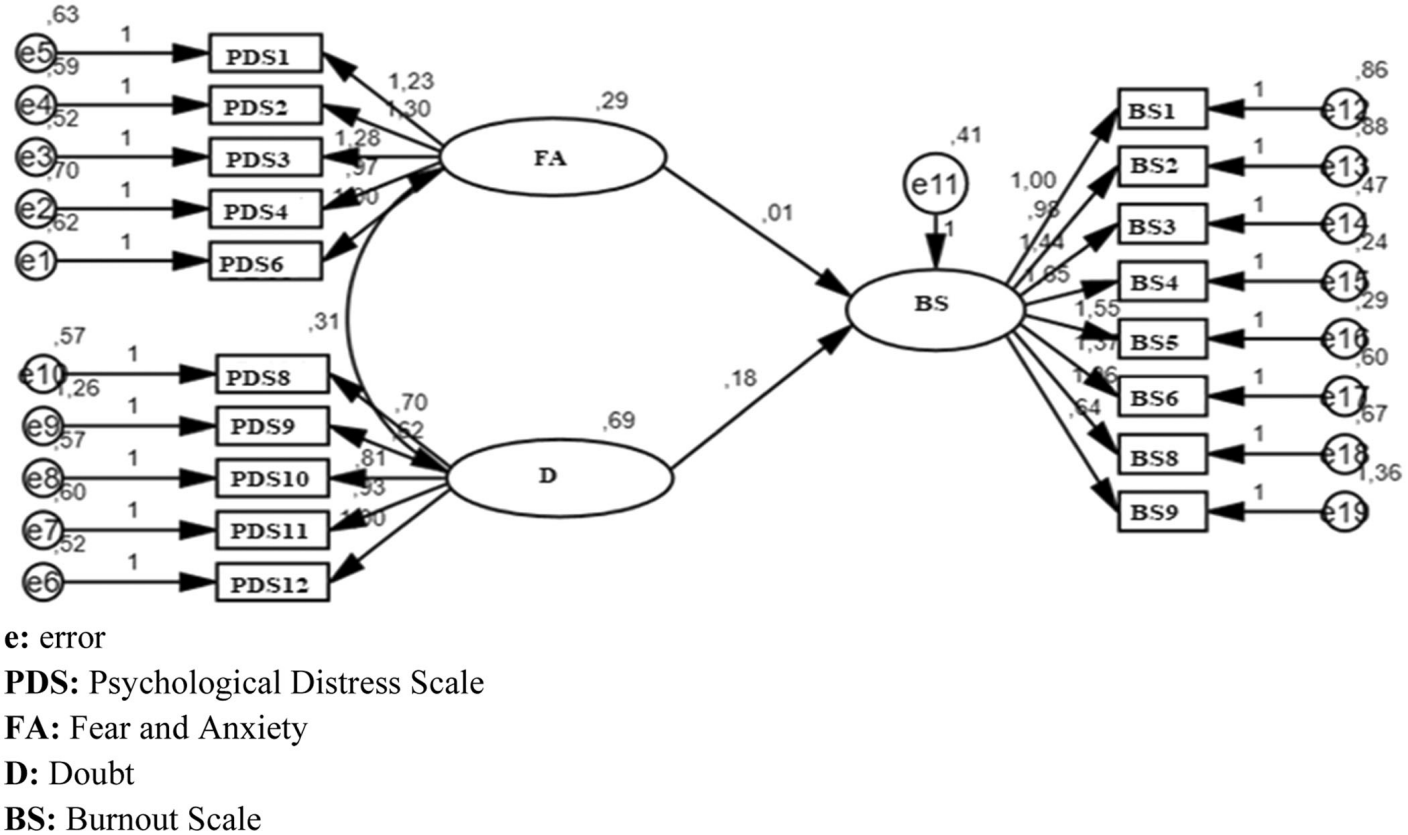
Structural equation model for the psychological distress and the burnout scales. e, error. PDS, psychological distress scale. FA, fear and anxiety. D, doubt. BS, burnout scale.

In the next stage of the study, a structural equation model (SEM) was used to test the effects of fear, anxiety, and doubt scores, which are the sub‐dimensions of the psychological distress scale, on psychological stress. Since the fit values of SEM, whose flowchart is given in Figure 1, were within acceptable limits, it was seen that sufficient evidence was provided that the model was structurally appropriate. According to the SEM results, when the relationship between the fear and anxiety sub‐dimensions of the psychological distress scale and the burnout scale was analyzed, no statistically significant relationship was found between these variables (*p* > 0.05). On the other hand, it was observed that the doubt sub‐dimension of the psychological distress scale affected the burnout scale of the participants (*β* = 0.178; *p* < 0.05). A one‐unit increase in the doubt sub‐dimension causes a 0.178‐unit increase in the burnout scale of the participants. When the R2 value obtained from the SEM model is examined, it is seen that 5.4% of the burnout scale is explained by the dimensions of the psychological distress scale.

Psychological distress and burnout scale scores were compared according to the demographic characteristics of the participants (Table 4). The fear and anxiety scores of the psychological distress scale (*p* = 0.000, 0.001), doubt scores (*p* = 0.000, 0.001) and the total score of the psychological distress scale (*p* = 0.000, 0.001) were found to be significantly higher in women than in men and in vaccinated participants than in unvaccinated participants.

**Table 4:** Comparison of psychological distress and burnout scale scores according to the demographic characteristics of the participants by Mann‐Whitney U test and Kruskal‐Wallis Test.

		Fear and anxiety	Doubt	Psychological distress	Burnout
**Gender** ^a^	Female	**18.672** ± **0.172**	**21.199** ± **0.257**	**39.871** ± **0.388**	**26.026** ± **0.413**
	Male	17.424 ± 0.296	19.300 ± 0.378	36.724 ± 0.588	24.200 ± 0.642
	*p*‐value	0.000	0.000	0.000	0.006
**Marital status** ^a^	Single	18.116 ± 0.179	20.774 ± 0.261	38.890 ± 0.394	26.329 ± 0.435
	Married	18.640 ± 0.281	20.312 ± 0.385	38.952 ± 0.597	23.753 ± 0.563
	*p*‐value	0.068	0.308	0.960	0.000
**Educational status** ^b^	Primary education	17.222 ± 0.698	18.000 ± 1.041	35.222 ± 1.492	22.722 ± 2.093
	High school	17.500 ± 0.524	19.696 ± 0.743	37.1960 ± 1.139	25.695 ± 1.180
	Associate degree	17.522 ± 0.738	19.435 ± 1.013	36.957 ± 1.624	24.869 ± 1.878
	Undergraduate	18.282 ± 0.194	20.927 ± 0.281	39.208 ± 0.427	26.079 ± 0.462
	Graduate	18.936 ± 0.325	20.696 ± 0.443	39.632 ± 0.679	24.192 ± 0.639
	*p*‐value	0.021	0.039	0.031	0.119
**Living place** ^b^	Province	18.269 ± 0.175	20.556 ± 0.242	38.826 ± 0.373	25.188 ± 0.391
	District	17.988 ± 0.315	20.452 ± 0.530	38.441 ± 0.755	26.417 ± 0.832
	Village	20.278 ± 0.636	22.833 ± 1.082	43.111 ± 1.611	27.889 ± 2.013
	*p*‐value	0.026	0.087	0.044	0.097
**Profession** ^b^	Health employee	19.146 ± 0.305	20.683 ± 0.436	39.829 ± 0.660	26.342 ± 0.721
	Small business	17.000 ± 1.567	20.417 ± 1.877	37.417 ± 3.213	19.250 ± 2.582
	Student	18.131 ± 0.200	20.856 ± 0.296	38.986 ± 0.444	26.804 ± 0.490
	Public sector	17.556 ± 0.747	19.000 ± 0.875	36.556 ± 1.516	21.222 ± 0.878
	Private sector	18.098 ± 0.562	20.171 ± 0.795	38.268 ± 1.176	20.951 ± 0.896
	Housewife	17.077 ± 0.540	19.731 ± 1.032	36.808 ± 1.389	25.500 ± 1.663
	Not working	19.235 ± 0.961	22.294 ± 1.236	41.529 ± 1.995	24.588 ± 1.984
	Worker	18.375 ± 1.487	17.750 ± 2.024	36.125 ± 3.259	20.625 ± 2.915
	*p*‐value	0.034	0.285	0.208	0.000
**COVID‐19 vaccine status** ^b^ **(test** + **)**	Positive	18.425 ± 0.153	20.789 ± 0.221	39.214 ± 0.333	25.480 ± 0.359
	Negative	15.933 ± 0.717	17.633 ± 0.849	33.567 ± 1.478	25.267 ± 1.527
	*p*‐value	0.001	0.001	0.001	0.973

a Mann‐Whitney U.

b Kruskal‐Wallis.

The fear and anxiety scores of the psychological distress scale were found to be significantly higher in graduates than in those with other levels of education, in those living in villages than in those living in other living places and in the unemployed compared to other occupational groups (*p* = 0.021, 0.026, 0.034). The psychological distress scale scores were found to be significantly higher in graduates than in those with other levels of education, and in those living in villages than in those living in living places (*p* = 0.031, 0.044). The doubt score of the psychological distress scale was found to be significantly higher in undergraduates than in those with other levels of education (*p* = 0.039).

Burnout scale scores were found to be significantly higher in women than in men, in singles than in marrieds, in those with chronic diseases than in those without, and in those with positive COVID‐19 posttest status compared to those with negative status (*p* = 0.006, 0.000, 0.046, 0.013). Burnout scale scores were found to be significantly higher in students than in other professional groups and in those with low income status compared to others (*p* = 0.000).

## 4 Discussion

Studies on anxiety, fear, and stress have been reported in many parts of the world during past pandemics and crises [11]. Fear, anxiety and doubts related to the COVID‐19 pandemic cause various mental health problems such as anxiety and psychological disorders [12]. The COVID‐19 pandemic, which has created a panic effect all over the world, has deeply affected our country. In this study, pandemic‐related psychological effects of individuals and related factors were examined during the COVID‐19 process.

According to the results of the study; fear and anxiety, suspicion, psychological distress, and burnout scores of female participants are higher than male participants (Table 4). In other studies examining the levels of fear and anxiety during the pandemic process, it was observed that women had higher levels of fear and anxiety than men [13, 14, 15, 16, 17, 18]. This may be attributed to the fact that women have lower psychological resilience and being a woman is a risk factor for various mental disorders. One of the reasons for women's higher fear and anxiety scores may be their relationship with gender roles [19, 20]. Since women are more comfortable in expressing distress, women may have higher anxiety scores [21]. In addition, while women express their emotions easily, men tend to suppress their emotions and appear strong [14]. Similarly, in a study conducted by Yazıcı Çelebi, it was determined that women were more affected by the pandemic process than men [22]. Similarly, a study conducted in Belgium stated that women have a higher risk of experiencing psychological distress [23].

It was found that the scores of fear and anxiety, suspicion and psychological distress were similar according to the marital status of the participants (Table 4). Bayülgen et al. found no significant difference between marital status and anxiety score during the pandemic process, similar to our study [24]. It was found that burnout scores of singles were higher than married individuals. The fact that married individuals have a partner to share their financial and moral burdens may cause them to think that they will not have to struggle alone with the problems that may develop.

The fear and anxiety scores of undergraduate and graduate graduates were higher than those of participants with other educational levels (Table 4). In a study conducted by Pak Güre et al. in Turkey, similar to our results, it was stated that the fear of getting COVID‐19 increased in individuals with higher education level [13]. Suspicion and psychological distress scores of primary school graduates are lower than other educational levels. Many studies have shown that the level of fear decreases as the level of education increases [16, 25]. However, we think that a similar result has not been found in Turkey because the Ministry of Health provides simple and understandable messages through social media and television to facilitate the understanding of the epidemic and to reduce unnecessary panic and stress. In addition, we think that highly educated people may tend to be more concerned about the pandemic, possibly due to their high self‐awareness about health [24]. Burnout scores of the participants were similar regardless of their level of education. In a study conducted in Turkey, it was stated that there was no significant difference between education status and fear, anxiety, and psychological stress scores [14].

In terms of living space; Fear, anxiety and psychological distress scores of participants living in villages are higher than participants living in other places (Table 4). The concerns of people living in rural areas about not being able to reach health institutions immediately, the low number of expert health professionals in these regions, and the concern about not being able to access effective treatment in case of serious illness may cause the scores of participants living in rural areas to be higher. Doubt and burnout scores were similar regardless of where participants lived. In the study conducted by Bakioğlu et al. in Turkey, it was shown that there was no significant difference between the place of residence and fear, anxiety and psychological stress scores [14].

Fear and anxiety scores of unemployed people and healthcare workers are higher than those working in other professional groups (Table 4). The higher scores of the unemployed may be due to the fact that these individuals think that they will be financially helpless and unable to access effective treatment if they contract the disease. There are studies in the literature indicating that the COVID‐19 pandemic has more psychological effects on healthcare workers than on the general population [26, 27, 28]. The high scores of healthcare professionals may be due to their concerns about being infected, contaminating family members, getting serious illness, and their higher awareness than the general population about serious illness, morbidity and mortality. When burnout scores are examined, students' scores are higher than other professional groups. Students' burnout scores may have been found to be higher due to reasons such as the uncertainty of the process, closure of schools, anxiety about the future, concern about falling behind academically and, accordingly, fear of being unemployed in the future. Participants' doubt and psychological distress scores were similar in all professional groups.

In terms of COVID‐19 job loss; participants' fear and anxiety, doubt, psychological distress, and burnout scores are similar. We think that the scores related to job loss were found to be low because most of the participants in our study were public sector employees and students.

Regardless of the income level of the participants, fear and anxiety, doubt and psychological distress scores are similar. Burnout scores of participants with income levels below 2000 TL are higher than participants with other income levels. In a study examining the mental health of the general population during the COVID‐19 pandemic in Japan in 2020, it was found that financial fragility during the epidemic affected the mental health status of the general population, especially the working‐age population [29].

When the participants were examined for the presence of chronic disease, fear and anxiety, doubt, and psychological distress scores were found to be similar. The burnout score of participants with chronic diseases is higher than those without. Jafri MR et al. In the study conducted by, COVID‐19 patients with comorbidities showed more psychological distress than those without comorbidities [30]. Participants with chronic diseases may think that if they are infected with COVID‐19, they will have a more severe disease, and perhaps the risk of death is higher than others.

In terms of COVID‐19 testing status; participants' fear and anxiety, doubt, psychological distress and burnout scores are similar. In terms of COVID‐19 latest test status; participants' fear and anxiety, doubt, and psychological distress scores are similar. However, the burnout scores of participants whose COVID‐19 posttest result was no were higher than those whose posttest result was yes. This may again be due to the uncertainty of the process and disease progression and the need for the person to continue quarantine and additional measures.

In terms of COVID‐19 vaccination status; Among vaccinated participants, those who tested positive had higher fear and anxiety, suspicion and psychological distress scores than those who tested negative (Table 4). The situation of being infected despite being vaccinated may have worried individuals. Regardless of COVID‐19 test result, participants who received the COVID‐19 vaccine had similar burnout scores.

According to the SEM results, it was observed that the suspicion sub‐dimension of the psychological distress scale affected the participants' burnout scale (Figure 1). A one‐unit increase in the suspicion sub‐dimension caused a 0.178‐unit increase in the participants' burnout scale. In a study conducted in Turkey, similar to our study, it was stated that there was a significant relationship between COVID‐19‐related psychological distress and COVID‐19‐related burnout, and that as the participants' psychological distress increased, the feeling of burnout also increased [31]. Job losses, disruptions in education, suspicion of infection and concerns about the future that emerged with the pandemic may have caused this situation.

## 5 Conclusion

Since stress and psychiatric disorders are more common during epidemic periods, especially in individuals with high anxiety, fear, and anxiety, long‐term psychological interventions need to be developed and implemented to protect the mental health of societies during these periods. Individuals should be informed by governments in a simple and understandable way to prevent panic and fear on issues related to the contagiousness, lethality, protection methods and vaccination of the agent. This study reports higher rates of psychological involvement in certain groups in the general population. These risk factors include being a woman, being single, unemployed, a student, a healthcare worker, living in a village, and having a chronic disease. Although these results require long‐term and more detailed observation, they confirm the necessity of preventive interventions to protect the mental health of the population in general, and especially of groups with these risk factors. We think that this study will be a useful guide for planning interventions to alleviate individuals' mental health problems in future epidemic/pandemic situations.

## 6 Limitations

Since the study was single‐centered, the number of participants who were public employees and students was high. Therefore, the results cannot be generalized to groups in the private sector and rural areas. Psychological impact and stress are based more on personal feelings. For this reason, the anxiety, fear, and stress levels reported by the participants may not be compatible with an objective assessment made by mental health experts. The majority of participants were public sector employees and students. For this reason, the results regarding job loss anxiety cannot be generalized. Due to the relatively small sample size in this study, we were not able to conduct a multifactor analysis on factors affecting COVID‐19‐related psychological distress. Additionally, it did not collect any information regarding participants' past medical psychiatric disorders as part of the study. Therefore, we think that more generalizable results can be achieved with more comprehensive examinations with larger sample groups in future studies. Additionally, this study examined immediate psychological effects during the pandemic, but did not address long‐term mental health outcomes. Longer‐term studies are needed to further assess the lasting effects of the pandemic on mental health.

## Author Contributions

**Bahar Ürün Ünal:** conceptualization, data curation, formal analysis, funding acquisition, investigation, methodology, project administration, resources, software, supervision, validation, visualization, writing – original draft, writing – review and editing. **Neslihan İyit:** data curation, visualization. **Yunus Akdoğan:** data curation, visualization. **Burcu Gök Erdoğan:** formal analysis, investigation, project administration, resources, software, supervision, writing – original draft, writing – review and editing. **Yüksel Duygu Altıparmak:** funding acquisition, resources, supervision, validation, writing – review and editing.

## Conflicts of Interest

The authors declare no conflicts of interest.

## Data Availability

All data generated and analyzed during this study are included in this published article.
